# Using Home-Cage Monitoring to Determine the Impact of Timed Mating on Male Mouse Welfare

**DOI:** 10.3389/fnins.2022.786652

**Published:** 2022-02-23

**Authors:** Joanna L. Moore, Eloisa I. Brook

**Affiliations:** GlaxoSmithKline, Brentford, United Kingdom

**Keywords:** mouse welfare, timed mating, home cage monitoring, laboratory mice, activity patterns

## Abstract

Some of our breeding programs include the use of Prm1 male Homozygous mice which are naturally sterile. This removes the need to use vasectomized males to induce pseudopregnancy in female mice. These males can be kept for up to 9 months and are housed with a companion female. During the timed mating period the companion female is replaced with a new female. This procedure can occur at regular intervals causing a significant increase in cage activity; one of our objectives was to determine whether this was as a result of timed mating. We wanted to investigate the disruption caused to mice during the day of the swap and how long it would take for the cage activity to return to pre-replacement baseline levels. We hypothesized that this impact would be reflected as a significant increase in cage activity, which in itself may not be a result of a negative experience but the potential of repeated disruption to their activity pattern should be considered. We used a well-known home-cage monitoring system to assess changes to the activity pattern in cages when a companion female is replaced. Data from our initial study showed that in the 2-h period after the female is replaced there is a significant increase in cage activity compared to the same time frame on the previous day. In the subsequent study, where no cage change occurred, an increase in activity was also observed when females were replaced; this returned to baseline after approximately 4 h. Prolonged activity during the rest period of mice (over 2 h) could lead to them being fatigued during their active period; therefore, as a refinement we propose that timed matings be performed later in the day, at a time when the animals are active.

## Introduction

The use of sterile male mice to induce pseudopregnancy in female mice assigned for the implantation of embryos is a vital component in the production of Genetically Altered Animals (GAA). Usually the male is vasectomized, which is a surgical procedure under general anesthesia, with time required for the male to recover and the spermatozoa count to drop to zero. One refinement to GAA production has been the development of genetically sterile mice, in particular the double transgenic Protomine1 (Prm1) strain of mouse. The male mice born with the dominant Prm1-EGFP transgene were found to be naturally sterile and can be used as a replacement for vasectomized mice (Haueter et al., 2010). These GAA mice have successfully replaced the need to use vasectomized male mice for generations of pseudo pregnant females in our facility. As the male mice utilized to induce pseudopregnancy cannot subsequently be re-housed with other males due to fighting, these mice are individually housed for up to 9 months depending on their breeding capability. Although male mice are not generally considered to be social animals there is evidence that shows they prefer to be housed with conspecifics (Goldsmith et al., 1978; Van Loo et al., 2002; Kalliokoski et al., 2014). In a breeding colony, fighting between a male: female pair or trio made up of one male and up to two female mice is less common, indicating that the sterile males may benefit from having a female companion over a male companion. Following on from work carried out by the originator of this model, each of our cohorts of sterile male mice have a companion female housed with them to reduce anxiety associated with long term single housing (Kalliokoski et al., 2014). When the male is required for the breeding program the companion female is replaced with a new female in the morning. The new female mouse is left in the cage for at least 24 h, or until a copulatory plug is observed in the vagina, indicating that mating has occurred.

We cannot be certain by visual observations alone what the duration and amount of disruption this intervention will cause in a mouse cage. Some of the reasons for this are: if there is an observer in the room it can lead mice to become more active than if they were undisturbed and if direct observations are used (where a person is in the room monitoring cages) there will undoubtedly be some subjectivity and or human error. The solution of a video recording can reduce this; however, it is very time consuming to review footage on a cage by cage basis.

To investigate the disruption caused to mice during the day of the replacement and how long it would take for the cage activity to return to baseline in an objective way, we housed mice in the Digital Ventilated Cage (DVC^®^) system, Tecniplast S.p.A, which is a non-intrusive mouse activity tracking system. This enables us to monitor mice without the need to use a video camera, subcutaneous microchip or have a telemetry device surgically implanted. Each cage position on the rack has a sensor board underneath it, containing 12 electromagnetic sensors to continuously track and monitor spontaneous mouse activity (Iannello, 2019) i.e., mouse activity without human intervention. The cage sits on the runners in the same way as any standard Individually Ventilated Cage (IVC) would, so there is no disturbance to the animals. The individual sensor board sends data generated by the cage occupants to a Master computer, which has a temporary data storage area and sends the data directly into a cloud storage platform from where it can be downloaded. Each time a mouse moves over an electrode, the mouse activates a signal which is sent to the Master computer, these activations form the “Animal Activity Index” which is a normalized measurement assuming values between 0% (no sensors activated) and 100% (all electrodes activated simultaneously). The summary measure over the cage is the Average Activity Index (AAI); calculated as the average of all twelve electrode activations per minute. When we discuss cage activity in this paper we are referring only to the AAI.

Other studies have shown that procedures involving cage changing handling or the transfer of the animals disrupts their basic activity pattern and induces changes that appear to impact them over an extended period of time (Gray and Hurst, 1995; Lim et al., 2019; Pernold et al., 2019). We wanted to investigate the disruption to cage activity during the day of the female exchange and how long it would take for the activity to return to baseline. We initially carried out a Pilot study with a small cohort of mice to investigate whether timed mating activity is masked by cage changing, followed by a subsequent (Main study) investigation of female replacement as part of timed mating. We hypothesized that the impact of mouse activity in the cage is significantly increased when a companion female is replaced with a new female.

**FIGURE 1:**
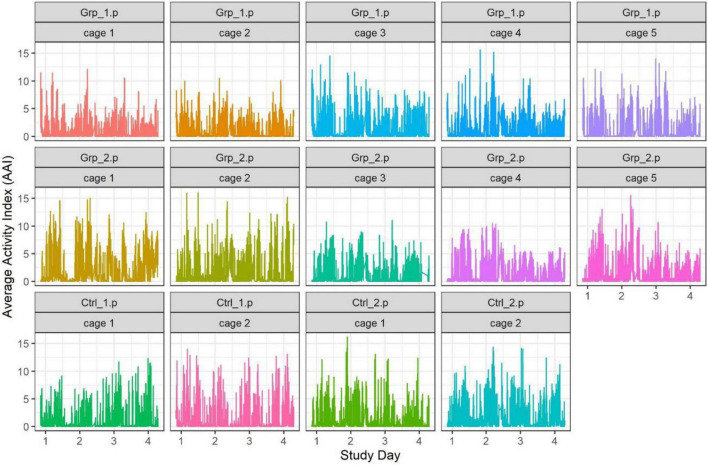
Graph showing individual cage activity during the Pilot study, each point represents the activity of a cage at every minute of the study.

The exploratory data analyses from these studies highlighted that there was a lot of variability between cages (including within groups) across each 24 h time frame (see Figure 1). In order to understand any changes in cage activity we decided to focus on specific 2-h time intervals. The 2-h time intervals we discuss here are when the mice are active after a cage is moved or opened [08:00–10:00 h (Main study only) and 10:00–12:00 h], when the mice were inactive (12:00–14:00 h) and, when the mice are active directly after the lights went out (20:00–22:00 h).

### Ethics Statement

All studies were ethically reviewed and carried out in accordance with Animals (Scientific Procedures) Act 1986 and the GSK Policy on the Care, Welfare and Treatment of Animals.

## Materials and Methods

All of the mice were bred at the establishment and housed in GM500 mouse cages (Tecniplast S.p.A) with a conventional wire lid. We included all available pairs of Prm1 males with their companion females and the CD1 females used as part of colony management. We had 14 Prm1 males and 14 CD1 female companions (bred at the establishment) and 10 CD1 females (Charles River Laboratories, United Kingdom) used as replacement females in the Pilot study and 20 Prm1 males, 20 CD1 companion females and 10 CD1 (Charles River Laboratories, United Kingdom) replacement females in the Main study. The male weights ranged between 33–56 gm and the female weights between 28–37 gm at the start of the Pilot study (see Supplementary Table 1).

Mice were transferred from the breeding room to the animal room. And on arrival the mice were health checked carefully and acclimatized for 7 days prior to the start of each study in XT GM500 Digitally Ventilated Cage (DVC^®^) (Tecniplast S.p.A, Italy) made from a clear Polypropylene plastic base with a stainless-steel bar lid, the internal measurements of the cage are 35.5 cm × 17.5 cm × 12 cm. The DVC^®^ was placed along the side wall near the rear of the room. A health monitoring program for mice was in place and excluded infectious agents described in the FELASA guidelines (Berard et al., 2014). For identification and randomization purposes all the females in these studies were ear marked prior to the start of the study. All new female mice were group housed in groups of four on an IVC rack in the room (prior to being introduced to the male) and established male/female pairs were housed on the DVC^®^ rack. When the companion female mice were removed from the cage, they were group housed with three other companion females on the IVC rack in the same animal holding room. The IVC rack was located along the side wall next to the DVC^®^ both were connected to a SmartFlow^®^ Air Handling Unit (Tecniplast S.p.A) located centrally between them.

All cages had 10 mm depth of Lignocel BK8/15 (IPS) bedding, a mouse igloo measuring 10 cm diameter × 6 cm high, and an aspen wood chew block 50 mm × 10 mm × 10 mm long (Datesand, United Kingdom) with a Lignocel Large Wood Wool disk (IPS, United Kingdom) and a mouse Mini Fun Tunnel 76 mm × 38 mm circumference (LBS, United Kingdom). Cages were changed in line with study protocol; where the mice are removed from the home cage and placed in a fresh cage with fresh identical bedding and fresh enrichment items.

Mice were fed *ad libitum* with 5LF2 irradiated rodent diet (IPS) *via* food hoppers at the rear of the cage, and *ad libitum* animal grade reverse osmosis filtered, and UV treated drinking water *via* bottles attached to the front of the cages. Food and water levels were monitored by the staff and the DVC^®^ system. After cage changing all enrichment remained with the animals for the duration of each study. Throughout the facility there was piped radio tuned into a local commercial station during the light phase. The light cycle was on a 14:10 light: dark with light phase from 06.00–20.00 h and a gradual increase or decrease of lighting over a 10-min dawn: dusk period. The room temperature was 21–24°C, humidity was 55% ± 10%, with 20 air changes per hour in the room and 75 air changes per hour in the DVC^®^ and IVC.

### Study Methods

We used established male: female pairs of Prm1 mice for both studies included in this research project. Each pair of mice had been together for at least 1 month. Given the studied effects on disruption to animal activity (Pernold et al., 2019) and physiologic parameters (Balcombe et al., 2004) from husbandry procedures including cage changing, we needed to be confident that any change in cage activity was related to female replacement alone. Therefore, the cages in the groups that were not subjected to the companion female being replaced, were removed from the rack and the companion picked up and placed back into the home cage after 3–5 s, by doing this we could be sure that any increase in activity would be directly related to the new female. We refer to this a sham replacement.

In order to pre-empt any sensitivity of the system to changes in the weights of the animals that could affect the AAI, the new females were weight matched to be as close as possible to the companion females.

#### Pilot Study

We included 14 established pairs of Prm1 male mice with their Wildtype companion females. A further 10 CD1 age matched new females were included as replacements for the companion females. Cages of animal pairs were randomly allocated to one of four groups as follows:

- Grp_1.p: Female replacement and no cage change (5 pairs/cages).
- Grp_2.p: Female replacement with a new female and a cage change (5 pairs/cages).
- Ctrl_1.p: Sham replacement and no cage change (2 pairs/cages).
- Ctrl_2.p: Sham replacement, and cage change (2 pairs/cages).

The study ran over a 5-day period; however, we focus on the data from Day 2 to Day 4 to have two 24 h periods of uninterrupted data for comparison. Day 3 was the day that the replacement/sham replacement/cage change occurred. The study ended early in the morning on Day 5, see Table 1 for full study design.

**TABLE 1:** Shows the group information for both studies and the time the staff were in the room to change and/or replace the females.

Study	Study Duration (days)	Group Name	Number of Cages	Factor(s) of Interest	Time Frame of Cage Interventions	Study Day of female replacement
Pilot Study	Five	Grp_1.p Ctrl_1.p Grp_2.p Ctrl_2.p	5 2 5 2	Female Replacement/No Cage Change Sham Replacement/ No Cage Change Female Replacement/Cage Change Sham Replacement/ Cage change	09:30 h–10:00 h	Three
Main Study	Ten	Grp_ 1.m Grp_2.m	10 10	Female Replacement Sham Replacement	07:00 h–08:00 h	Six

*All cages started and ended with a male and a companion female, the table shows study duration and the factors tested.*

Home cage activity data was collected from the DVC^®^ for the entire study (see Figure 1), however, as previously discussed we focused on the following timeframes: 10.00–12.00, 12.00–14.00 and 20.00–22.00 h.

**FIGURE 2:**
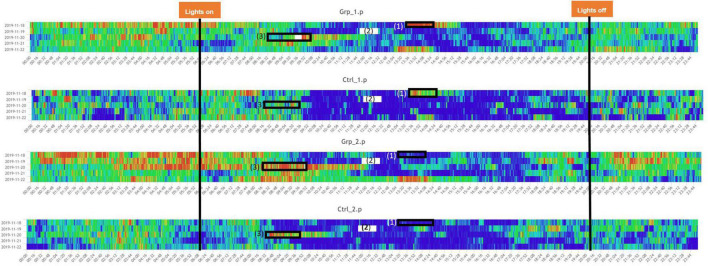
Heatmaps across the full study period for all groups. (1) Indicated period when the mice in Grp_1.p were cage changed, and Ctrl_1.p were removed and put back in their original cage, the mice in Grp_2.p and Ctrl_2.p were sham cage changed. (3) Indicated period when the companion female was replaced with a new female for Grp_1.p and Ctrl._1.p had a sham replacement, both without a cage change, Grp_2.p cages had the companion female was replaced with a new female and the cages were changed, and Ctrl._1.p had a cage change only. (2) Indicates a period when there was a system error and no data was recorded. Note: time aggregation is 1-min intervals.

We defined the baseline as either the average activity on the day prior (Day 2) or the day after (Day 4) the female replacement. The cage change was carried out at the same time of day as the female replacement/sham replacement to enable us to determine if the effect of cage changing would mask activity caused by the female replacement/sham replacement. During the Pilot study we saw a significant spike in activity (see Figures 1, 2) as a result of cage changing, which has also been reported by Pernold et al. (2019), as a result we removed the potentially confounding effect of cage changing for the Main study.

#### Main Study

We included 20 established pairs of Prm1 mice and 10 CD1 weight matched females were included for the new female cohort. Cages were randomly allocated to one of two groups as follows:

- Grp_1.m: Female replacement (10 pairs/cages).
- Grp_2.m: Sham replacement (10 pairs/cages).

We decided to extend the duration of the study to allow us to observe several days of animal activity both before and after the “swap” day. Therefore, this study ran over a 10-day period, with the female replacement occurring on Day 6 the study terminating in the morning of Day 10. As before, and similar to what we had used in the Pilot study we focused our analysis on four 2 h-time frames where the mice were either usually active or usually inactive prior to any intervention: 08:00–10:00 h, 10:00–12:00 h, 12:00–14:00 h and 20:00–22:00 h. We compared the average activity of each of these timeframes on Day 6 (female replacement day) against the average of all previous days (Days 1:5), and then also against the average of all subsequent days (Days 7:10). This was to determine how much the activity for each cage had increased or decreased on the day of female replacement compared to the average pre and post replacement-day data. We defined the baseline as either the average activity of all previous days (days 1:5) or all subsequent days (days 7:10).

An overview of each study plan can be seen in Table 1.

### Statistical Methods

The cages (considered as the experimental unit) were randomized to the groups by stratifying on the companion female weight when it was available or by doing a simple randomization when it was not. All analyses, including randomization, were carried out in R (version 3.02 or above) *via* R- Studio.

The data we have analyzed is the Average Activity Index (AAI), which is collected every minute for each cage, including during the periods of cage changing and animal welfare checks. In order to minimize any over-inflation of results and given the spikes that were observed during these events, we removed the data where there were events such as cages being removed for observations and replacements.

Initially a traditional ANOVA and *T*-test was carried out on the data for specified time frames, in addition the data from the Main study was then analyzed through functional data analysis (Morris, 2015). Each cage activity had a regularized Fourier series fitted, afterward undergoing a permuted *T*-test, with a correlation aware multiplicity *p*-value adjustment (Westfall and Young, 1993).

#### Pilot Study

We used a two-way ANOVA to compare between groups to see the effect of replacing the female companion mouse and cage changing, and to see if there was an interaction between them. We focused on the activity data generated in three 2-h time frames (10:00–12:00 h, 12:00–14:00 h and 20:00–22:00 h) on the day of the female replacement (Day 3) compared to Day 2 and Day 4.

#### Main Study

We ran a *T*-test on the change from baseline measurement for each time interval to investigate the effect of the female replacement (Grp_1.m) on cage activity compared with a sham replacement (Grp_2.m). We ran this *T*-test at each of the following specific timeframes: 08:00–10:00 h, 10:00–12:00 h, 12:00–14:00 h and 20:00–22:00 h. Function on scalar regression was also applied to the full dataset 24 h before and after replacement using the FDA package in R. The use of functional data analysis was to obtain a more precise estimate of the “return to baseline” measurement after the females are replaced.

## Results

In the Pilot study females were replaced between 09:30 and 10:00 h, the comparison of Day 3 vs. Day 2 (pre-female replacement) showed a significant increase in activity for the 10:00–12:00 h time frame, due to both Female Replacement and Cage Change (see Table 2). There was no significant difference in activity of female replacement or cage activity for the timeframes 12:00–14:00 h and 20:00–22:00 h, see Supplementary Table 2 for a complete set of results.

**TABLE 2:** Statistically significant results of the two-way factorial ANOVA from the Pilot study and from the *T*-Test from the Main study.

Study	Comparison day	Analysis time	Effect	Sum of squares	*P* value
Pilot Study	**Day 3 vs. Day 2**	10:00–12:00 h	Female replacement	6.174	**0.006**
			Cage change only	2.325	**0.06**
Main Study	**Day 6 vs. Days 1:5**	10:00–12:00 h	Female replacement	9.516	**0.0198**
	**Day 6 vs. Days 7:10**	10:00–12:00 h	Female replacement	3.568	**0.036**
		12:00–14:00 h	Female replacement	0.453	**0.023**

*For a complete set of results see Supplementary Table 2.*

In the Main study females were replaced between 07:00–08:00 h and the *T*-tests showed, in the cages where females were replaced (Grp_1.m), there was a significant difference in activity in the time frame comparison for 10:00–12:00 h when compared to the average activity of previous days, and for the comparisons of the time frame for 10:00–12:00 h and 12:00–14:00 h when compared to subsequent days (see Table 2).

Function on scalar regression tests showed that the cage activity in Grp_1.m (where females were replaced) had higher activity peaks and returned to baseline ∼ 4 h post intervention (Figure 8) compared to the sham replacement in Grp_2.m where there was no significant increase in activity compared to pre-replacement.

### Pilot Study

**FIGURE 3:**
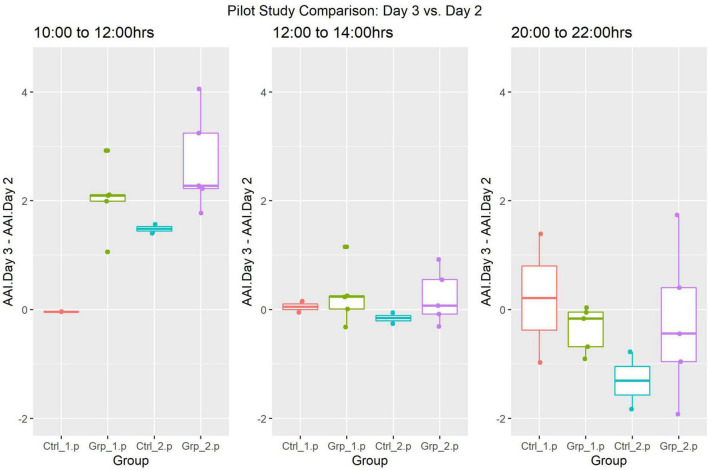
Pilot Study boxplots of the Average Activity Index for all groups represented as Day 2 (pre-replacement/baseline) subtracted from Day 3 (replacement day).

The Two-way Factorial ANOVA carried out compared the activity on Day 2 and Day 3, at each of the three-time frames (Figure 3). The statistically significant differences between the control groups (Ctrl_1.p and Ctrl_2.p) and between Grp_1.p and Grp_2.p are seen in (a), and we see in (b) how we are no longer able to see differences between groups at 12:00–14:00 h. For a full set of statistical results see Supplementary Table 2.

**FIGURE 4:**
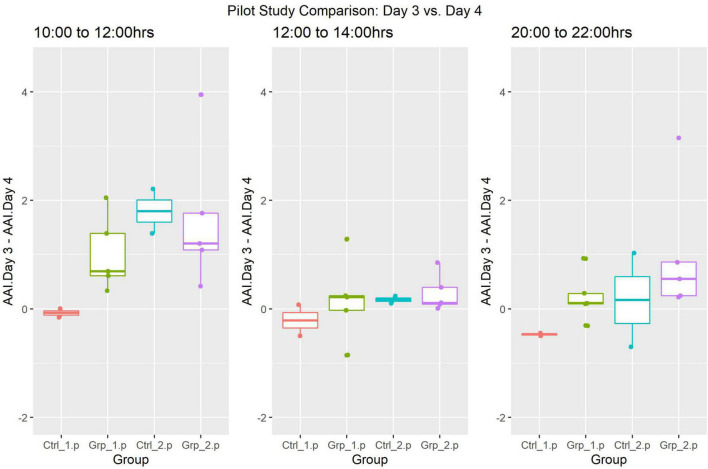
Pilot Study boxplots of the Average Activity Index for all groups represented as Day 4 (post-replacement/baseline) subtracted from Day 3 (replacement day).

ANOVA was also used to compare the activity between Day 3 and Day 4 (Figure 4), however, there were no statistically significant differences in any of the time frames when Day 3 was compared to the same time frames in Day 4.

### Main Study

**FIGURE 5:**
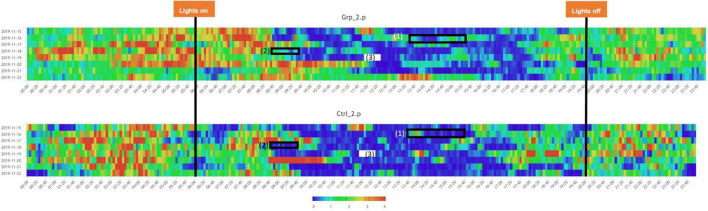
Heatmaps showing activity across full study period for both groups., (1) indicated period when the mice were cage changed causing a high peak in activity for the mice. (2) indicated period when the companion female was replaced with a new female (Grp_1.m) or sham replaced (Grp_2.m). Time aggregation is 5-min intervals.

**FIGURE 6:**
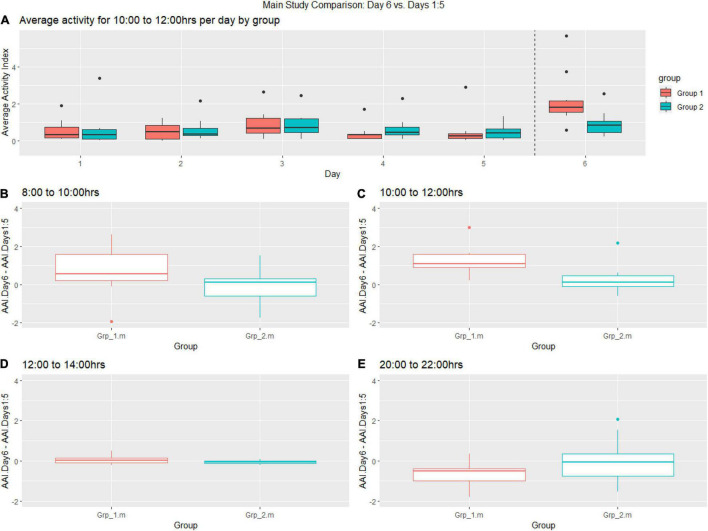
**(A)** Boxplot showing pre-female replacement (Days 1:6) average activity of groups per day between 10:00–12:00 h, **(B–E)** Change of baseline for Grp_1.m (female replacement) and Grp_2.m (sham replacement) between 08:00–10:00, 10:00–12:00 h, 12:00–14:00 h and 20:00–22:00 on the day of replacement compared to previous days.

In theMain Study, we reviewed the data and generated a heat map of mouse activty across theMain Study Period (see Figure 5). We observe that the cage activity at the typically quiet time 10:00–12:00 h is very similar in all the days leading up to intervention Day 6 and, we notice a significant increase for Grp_1.m on Day 6 as compared to previous days and Grp_2.m (Figure 6A). We observe an increase in activity for Grp_1.m in 08:00–10:00 h (Figure 6B) and by 10:00–12:00 h (Figure 6C) this difference is statistically significant. Conversely, we see a reduction in activity for this group at the 20:00–22:00 h (Figure 6E).

**FIGURE 7:**
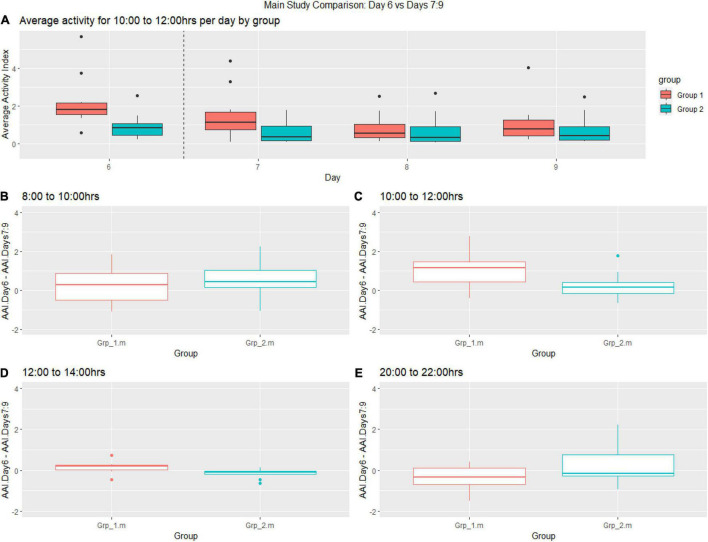
Graphs shows post female replacement (Day 6 = day of intervention and Days 7–9) activity. **(A)** Average activity from 10:00–12:00 h of groups per day. **(B–E)** Change from the baseline between **(B)** 08:00–10:00, **(C)** 10:00–12:00 h, **(D)** 12:00–14:00, **(E)** 20:00–22:00.

The post-female replacement analysis generally showed more variability in the activity data sampled between 08:00–10:00 h compared to other time frames. We observe a decrease in activity for Grp_1.m (female replaced) between 10:00–12:00 h for each day post-replacement (Figure 7A), where the activity for Grp_2.m is very stable. There was a statistically significant increase in activity between the groups during the 10.00–12.00 h time frame (see Figure 7C) and 12:00–14:00 h (see Figure 7D). As seen before there is a trend toward a reduction in activity for Grp_1.m (female replaced) in the time frame 20:00–22:00 h (see Figure 7E) compared to Grp_2.m.

There are of course limitations in analyzing the data in 2-h intervals as we have been doing until now, to complement this and use the full minute by minute data set, we decided to apply functional regression to explore if, with a more detailed data set, we were able to estimate with more precision any changes in activity between the groups after the replacement and for how long this was sustained.

**FIGURE 8:**
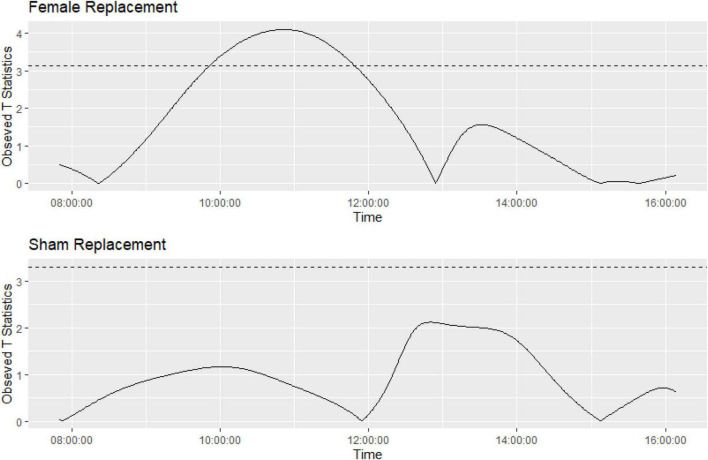
Graph showing the function on scalar regression permuted *t*-tests showing significantly different activity during the study timeframes for Grp_1.m **(top graph)** compared to Grp_2.m **(bottom graph)**. Horizontal line represents appropriate *p*-value adjustment (Westfall and Young, 1993), observed t-statistics above this are deemed significantly different.

Functional regression is a flexible approach to the modeling of data. The three main components of functional regression are the basis functions, replication, and regularization. In the context of animal activity, it would be appropriate to select a Fourier series with appropriate expansion as basis functions, as the animal activity may be assumed to be periodic. The replicates are the cages, and the fit of each cage is regularized to prevent overfitting. The functions produced to model activity within each cage have been smoothed into an overall function to model the activity for the pre-event and post-event activity. The activity after the event has been compared to the activity pre-event, using permuted *t*-tests with an appropriate *p*-value adjustment. For Grp_1.m, it appears that the activity returns to baseline after approximately 4 h, with Grp_2.m having no difference in activity compared to baseline. These results closely match the timeframes we had found as significantly different for Grp_1.m, and the overall conclusions are the same (see Figure 8).

## Discussion

Initially we investigated whether the impact of replacing a female would be masked during the cage change process as studies have shown that there is a significant increase in activity post cage change (Pernold et al., 2019). We found the increased spike in activity in the 2 h after the female replacement was conducted was much more significant than the actual disruption caused by cage changing (Table 2). However, cage change was still a significant effect within this time frame and therefore, we removed cage changing from the Main study where we wanted to investigate whether replacing females 6 days after cage changing would show higher activity than not replacing the females and how long it would take for the disruption to return closer to the pre-replacement baseline.

We found that there was an increase in activity across the cages where the female was replaced compared to when the companion female was briefly removed. From the *T*-Test analyses of the Main study, we inferred that across the study period it took 4 h for the activity levels to be similar to pre-replacement baseline activity levels across all the timeframes, we also tested the return to baseline more precisely using Functional regression and found the activity levels returned to baseline 4 h directly post intervention. Home Cage Monitoring gave us an insight into the impact of timed mating on the expected activity pattern of the study mice. We found a notable increase in activity during the light phase and an unexpected decrease in activity during the dark phase (see Figures 6, 7) as a response to female replacement, while not statistically significant it is an interesting finding that we would not have known if it were not for the DVC^®^, which is another benefit to having 24 h cage monitoring.

Understanding how our routines and procedures impact animal activity is important, especially if it is likely to have a negative effect on the animals and likely to be repeated with the same animal over a number of months such as timed mating for stud male mice. Interventions that are considered “non-invasive” such as cage changing (Rosenbaum et al., 2009; Pernold et al., 2019), or “sub threshold” such as timed mating, can cause significant spikes in activity which are not fully understood using a traditional scoring system based on cage side observation. For example, changes in activity are usually included in welfare score sheets for moderate to severe protocols. These score systems (Ullman-Cullere and Foltz, 1999) have been used as an objective assessment of changes in behavior and body condition which can indicate pain and distress in mice. This is an “in the moment” assessment which usually involves having to remove a cage from the rack. This disturbance would not give any meaningful observation data when trying to observe the response of mice to timed mating. Therefore, significant disruptions in the activity patterns of mice (Pernold et al., 2019), which could lead to increased levels of stress may be missed.

The importance of sleep in mammals has been demonstrated (Horne and McGrath, 1984; Tang et al., 2005; Palchykova et al., 2006; Colavito et al., 2013; Febinger et al., 2014) and the resulting stress associated with sleep disturbance could cause a confounding factor for experiments (Van Loo et al., 2002). Our study has indicated that timed mating may have an effect on the wellbeing of mice due to the potential disturbance in their activity pattern. The disruption in the activity pattern could lead to increase stress levels in male mice if they are used for repeated timed mating. While we can be confident the activity is as a result of replacing the companion female with a new female, we cannot be certain whether the activity is just the male, female or more likely it is a combination of both mice.

Our initial program of work was designed with a focus on the potential impact of timed mating on a male mouse, however, in doing so we missed out on observing the female’s response to their own different scenarios and whether there is any impact on their welfare. It would be interesting to follow the companion females and see how they react to being put in a new cage with other companion females and what happens when they are returned to the male. We postulated activity may be lower when she is returned to a familiar cage compared to if she were put in with an unfamiliar male in a new cage, especially if there are still smells and cues that are familiar. If, in addition, we included the impact on the new female before and after she has been with the male, this could give insight of how timed mating impacts all the mice involved.

Synchronizing our interventions to the activity pattern of mice may lead to improving their wellbeing (Febinger et al., 2014; Pernold et al., 2019). The impact of timed mating could be reduced if we carried out this procedure later in the afternoon as this is closer to the time that mice are more active. In the Pilot study the new female was left with the male for 5 days however, it should be noted that routinely the new female is removed after 24 h, but there are occasions when the initial mating is not successful, and females are left with the male for an extended period. This could lead to the male becoming accustomed to the new female, and when she is replaced with the companion female it becomes another disturbance in his natural activity pattern. Anecdotal observations from the research team were that mating was observed immediately after female replacement and continued for the duration of time that they were present in the room and was the most likely reason for the peak in activity, the use of a video camera would enable us to be certain that this observation was correct.

In conclusion this study has shown there is a potential impact on the welfare of male mice during timed mating as a result of the changes in their activity pattern. The impact of this intervention could be reduced if the timed mating occurred in the late afternoon when the mice are in the active part of their day.

## Data Availability Statement

The raw data supporting the conclusions of this article will be made available by the authors, without undue reservation.

## Ethics Statement

The animal study was reviewed and approved by GlaxoSmithKline, United Kingdom. All studies were ethically reviewed and carried out in accordance with Animals (Scientific Procedures) Act 1986 and in the GSK Policy on the Care, Welfare and Treatment of Animals.

## Author Contributions

JM devised the initial study, worked with the staff in the room carrying out the practical work, and wrote the first draft of the manuscript’s introduction and methods. EB helped refine the study design and method for the second study, oversaw the statistical analysis on the data, helped produce the results, and wrote the results. Both authors contributed to the discussion and editing and refining the whole manuscript to prepare it for submission.

## Conflict of Interest

JM and EB are employed by the company GlaxoSmithKline, Brentford, United Kingdom.

## Publisher’s Note

All claims expressed in this article are solely those of the authors and do not necessarily represent those of their affiliated organizations, or those of the publisher, the editors and the reviewers. Any product that may be evaluated in this article, or claim that may be made by its manufacturer, is not guaranteed or endorsed by the publisher.
