# Supplementary material for: Using Home-Cage Monitoring to Determine the Impact of Timed Mating on Male Mouse Welfare
Source: Front Neurosci. 2022 Feb 23;16:786652. doi: 10.3389/fnins.2022.786652 (PMC8904386; doi:10.3389/fnins.2022.786652)
Supplement: Supplementary file 1 [file Table_1.pdf]

Supplementary Table 1

Group	Count	Females	Males
Ctrl_1.p	2	30.7 (1.13)	49.8 (5.94)
Ctrl_2.p	2	32.85 (6.72)	53.25 (4.03)
Grp_1.p	5	34.54 (1.04)	39.12 (5.25)
Grp_2.p	5	32.38 (2.1)	43.8 (6.52)
Replacement Females	10	27.56 (1.56)	-
Grp_1.m	10	38.13 (4.88)	-
Grp_2.m	10	38.52 (3.65)	-
Replacement Females	10	31.42 (2.83)	-

Average and Standard Deviation weight by group for each study. N.B. The same males were used for both the Pilot and Main study. However, we only weight matched the females and therefore, did not weigh the males for the Main study.

Supplementary Table 2

Study	Comparison day	Analysis time	Source of Variation	Sum of Squares	P value
**Pilot Study**	**Day 3 vs. Day 2**	10:00-12:00hrs	Intercept	0.003	0.937
			Female replacement	6.174	**0.006**
			Cage change	2.325	**0.06**
			Female replacement: cage change	0.511	0.344
			Residuals	5.175	
		12:00-14:00hrs	Intercept	0.005	0.884
			Female replacement	0.066	0.601
			Cage change	0.044	0.666
			Female replacement: cage change	0.022	0.762
			Residuals	2.249	
		20:00-22:00hrs	Intercept	0.087	0.791
			Female replacement	0.455	0.548
			Cage change	2.308	0.192
			Female replacement: cage change	1.91	0.232
			Residuals	11.78	
**Pilot Study**	**Day 3 vs. Day 4**	10:00-12:00hrs	Intercept	0.012	0.914
			Female replacement	1.702	0.213
			Cage change	3.529	0.085
			Female replacement: cage change	1.043	0.323
			Residuals	9.286	
		12:00-14:00hrs	Intercept	0.09	0.595
			Female replacement	0.211	0.422
			Cage change	0.145	0.502
			Female replacement: cage change	0.049	0.694
			Residuals	2.996	
		20:0022:00hrs	Intercept	0.446	0.482
			Female replacement	0.684	0.387
			Cage change	0.403	0.504
			Female replacement: cage change	0.016	0.893
			Residuals	8.367	

**Main Study**	**Day 6 vs. Days 1:5**	08:00-10:00hrs	Female replacement	3.038	0.1455
		10:00-12:00hrs	Female replacement	9.516	**0.0198**
		12:00-14:00hrs	Female replacement	0.096	0.105
		20:00-22:00hrs	Female replacement	2.882	0.109
**Main Study**	**Day 6 vs. Days 7:10**	08:00hrs-10:00hrs	Female replacement	0.398	0.517
		10:00-12:00hrs	Female replacement	3.568	**0.036**
		12:00-14:00hrs	Female replacement	0.453	**0.023**
		20:00s-22:00hrs	Female replacement	3.177	0.064
